# Height indicates hematopoietic capacity in elderly Japanese men

**DOI:** 10.18632/aging.101061

**Published:** 2016-10-04

**Authors:** Yuji Shimizu, Shimpei Sato, Jun Koyamatsu, Hirotomo Yamanashi, Mako Nagayoshi, Koichiro Kadota, Takahiro Maeda

**Affiliations:** ^1^ Department of Community Medicine, Nagasaki University Graduate School of Biomedical Science, Nagasaki, Japan; ^2^ Department of Cardiovascular Disease Prevention, Osaka Center for Cancer and Cardiovascular Disease Prevention, Osaka, Japan; ^3^ Research and Clinical Center for Yusho and Dioxin, Kyusyu University, Fukuoka, Japan; ^4^ Department of Island and Community Medicine, Nagasaki University Graduate School of Biomedical Science, Nagasaki, Japan

**Keywords:** reticulocyte, hematopoiesis, height, elderly men

## Abstract

Previously, we reported that height is an indicator of the capacity of vascular repair in elderly men, especially hypertensive men. On the other hand, hemoglobin could act as a possible biochemical index of hypertension-induced vascular damage. However, no studies have clarified the correlation between height and hematopoietic activity. We conducted a cross-sectional study of 249 men aged 65-69 undergoing a general health check-up. Reticulocyte was used to evaluate hematopoietic activity. Because hemoglobin concentration should influence hematopoietic activity, analyses stratified by hemoglobin level were performed. Independent of known cardiovascular risk factors and other hematological parameters (white blood cell count), a significant positive correlation was seen between height and reticulocytes for total subjects and subjects with a high hemoglobin concentration (≥14.5 g/dL), but not in subjects with a low hemoglobin concentration (<14.5 g/dL). The standardized parameter estimates (β) were β=0.18, p=0.003 for total subjects, β=0.28, p=0.001 for subjects with a high hemoglobin concentration, and β=0.03, p=0.717 for subjects with low hemoglobin. Independently, height is significantly positively correlated with reticulocyte in elderly Japanese men, particularly in men with a high hemoglobin concentration. These results indicate that subjects with a short stature might have lower hematopoietic capacity than those with a high stature.

## INTRODUCTION

Several studies have reported an inverse relation between height and incidence or morality of cardiovascular disease [1–10].

Although it is believed that a decline in hemoglobin levels might be a normal consequence of aging, various studies have provided accumulating evidence that anemia reflects poor health and increased risk of poor outcomes in the elderly [11,12].

On the other hand, active hematopoietic (red) bone marrow, which plays an important role in hematopoie-sis, declines with age and is transformed into fatty (yellow) marrow from the periphery towards the axial skeleton [13]. Since the volume of bone marrow is smaller in subjects with a shorter stature compared to those with a taller stature, any reduction in the activity of hematopoietic bone marrow (hematopoiesis) could be of crucial importance for the former group.

However, no epidemiological studies clarifying the correlation between height and hematopoietic activity among elderly subjects have been conducted.

Previously, we reported that height indicates the capacity of vascular repair in elderly men with hypertension [14]. We also reported a significant positive association between hemoglobin and hypertension [15], hypertension-induced vascular damage [16], and atherosclerosis [17]. These studies indicate that hemoglobin level should act as an evaluator of the correlation between height and hematopoietic capacity, since hemoglobin level is an indicator of the necessity of vascular maintenance and hematopoietic activity.

In addition to the above, height is known to be significantly correlated with age in Japanese men [10]. Since the aim of our present study was to evaluate the influence of height on reduced capacity of hematopoietic bone marrow (hematopoiesis) with aging, we a conducted cross sectional study of elderly Japanese men within a narrow age range (65-69 years) who participated in a general health check-up in 2013-2015.

## RESULTS

No significant correlation was found between height and age in the present study population (simple correlation coefficient (r) =-0.11 (P=0.088).

The characteristics of the study population accounting for hemoglobin concentration are shown in Table 1. Subjects with a high hemoglobin concentration (≥14.5 g/dL) had significantly higher reticulocytes, WBCs, systolic blood pressure, diastolic blood pressure, BMI, and triglycerides compared to those with a low hemoglobin concentration (<14.5 g/dL).

**Table 1:** Characteristics of the study population

	High hemoglobin (≥14.5 g/dL)	Low hemoglobin (<14.5 g/dL)	p
No. of participants	122	127	
Age, years	67.5 ± 1.3	67.2 ± 1.3	0.118
Reticulocytes, ‰	12.13 ± 3.53	10.43 ± 3.36	<0.001
White blood cells, cells/μL	5739 ± 1360	5231 ± 1317	0.003
Systolic blood pressure, mmHg	137 ± 17	131 ± 18	0.003
Diastolic blood pressure, mmHg	82 ± 11	76 ± 12	<0.001
Body mass index (BMI), kg/m^2^	22.4 ± 1.8	21.9 ± 1.8	0.043
Serum HDL-cholesterol (HDL), mg/dL	57 ± 13	59 ± 15	0.425
Serum triglycerides (TG), mg/dL	114 ± 55	112 ± 106	0.043
Hemoglobin A1c (HbA1c), %	5.8 ± 0.7	5.6 ± 0.5	0.132
Serum aspartate aminotransferase (AST), IU/L	25 ± 10	24 ± 7	0.280
Serum γ-glutamyltranspeptidase (γ-GTP), IU/L	48 ± 46	41 ± 34	0.077
Serum uric acid (UA), mg/dL	6.0 ± 1.2	5.8 ± 1.2	0.324
Serum creatinine, mg/dL	0.83 ± 0.14	0.84 ± 0.15	0.355
Height, cm	164.3 ± 6.2	164.2 ± 4.9	0.900

Values are mean ± standard deviation.

Table 2 shows the simple correlation coefficient by simple regression analysis. Height showed a slight but significant positive correlation with reticulocytes for total subjects and subjects with a high hemoglobin concentration, but not for subjects with a low hemoglobin concentration (simple correlation coefficient (r) =0.13, p=0.047 for total subjects, r=0.21, p=0.019 for subjects with a high hemoglobin concentration, and r=0.02, p=0.810 for subjects with low hemoglobin).

**Table 2:** Simple correlation analysis of reticulocytes and other variables

	Total		High hemoglobin (≥14.5 g/dL)		Low hemoglobin (<14.5 g/dL)	
	r	p	r	p	r	p
No. of participants	249		122		127	
Age	−0.07	0.276	−0.16	0.087	−0.04	0.691
Systolic blood pressure	−0.04	0.577	−0.14	0.137	−0.04	0.691
Diastolic blood pressure	−0.06	0.376	−0.15	0.093	−0.09	0.336
Body mass index (BMI)	0.09	0.166	0.05	0.553	0.06	0.470
Serum HDL-cholesterol (HDL)	−0.08	0.221	−0.15	0.092	0.01	0.910
Serum triglycerides (TG)	0.22	0.001	0.26	0.004	0.15	0.092
Hemoglobin A1c (HbA1c)	0.01	0.892	0.05	0.617	−0.09	0.300
Serum aspartate aminotransferase (AST)	0.13	0.038	0.17	0.057	0.05	0.593
Serum γ-glutamyltranspeptidase (γ-GTP)	0.28	<0.001	0.36	<0.001	0.16	0.070
Serum uric acid (UA)	0.12	0.061	0.12	0.205	0.10	0.271
Serum creatinine	0.02	0.809	0.01	0.917	0.05	0.570
White blood cells	0.32	<0.001	0.42	<0.001	0.15	0.085
Height	0.13	0.047	0.21	0.019	0.02	0.810

r: simple correlation coefficient. TG, γ-GTP and serum creatinine are calculated in logarithm values.

**Figure 1:**
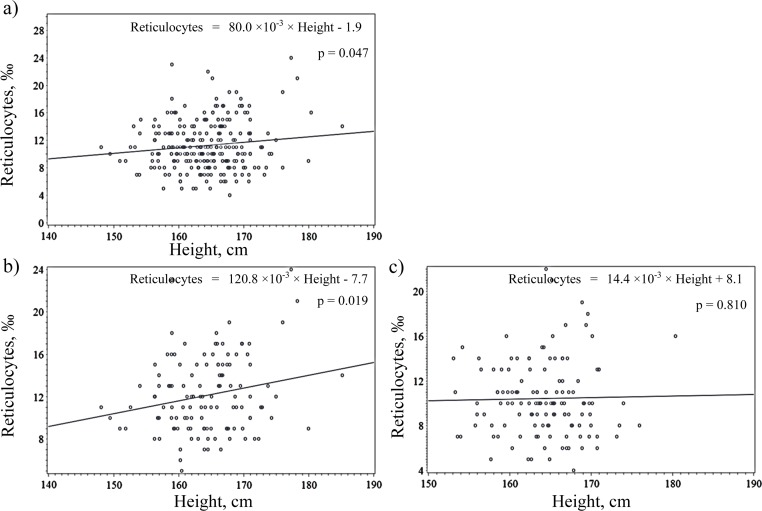
Simple linear regression analysis of reticulocytes and height among (**a**) total subjects, (**b**) subjects with high hemoglobin and (**c**) subjects with low hemoglobin.

From simple linear regression analysis, a linear correlation was observed between reticulocytes and height for total subjects and subjects with a high hemoglobin concentration, but not for subjects with a low hemoglobin concentration (Figure 1).

After further adjustment for known cardiovascular risk factors and other hematological parameters (white blood cell count), this correlation became slightly stronger, as shown in Table 3 (β=0.18, p=0.003 for total subjects, β=0.28, p=0.001 for subjects with a high hemoglobin concentration, and β=0.03, p=0.717 for subjects with a low hemoglobin concentration).

**Table 3:** Multivariable correlation analysis of reticulocytes and other variables

	Total			High hemoglobin (≥14.5 g/dL)			Low hemoglobin (<14.5 g/dL)		
	β	95% CI	p	β	95% CI	p	β	95% CI	p
No. of participants	249			122			127		
Age	−0.01	(−0.12, 0.11)	0.932	−0.08	(−0.23, 0.07)	0.282	0.010	(−0.18, 0.20)	0.917
Systolic blood pressure	−0.06	(−0.18, 0.06)	0.323	−0.12	(−0.28, 0.03)	0.127	−0.05	(−0.22, 0.14)	0.625
Body mass index (BMI)	0.06	(−0.05, 0.18)	0.276	0.07	(−0.09, 0.23)	0.399	0.08	(−0.10, 0.24)	0.419
Serum HDL-cholesterol (HDL)	0.01	(−0.12, 0.15)	0.827	−0.03	(−0.22, 0.15)	0.708	0.07	(−0.13, 0.25)	0.532
Serum triglycerides (TG)	0.13	(−0.004, 0.26)	0.056	0.12	(−0.07, 0.34)	0.190	0.13	(−0.07, 0.29)	0.232
Hemoglobin A1c (HbA1c)	−0.02	(−0.14, 0.10)	0.775	0.02	(−0.13, 0.16)	0.849	−0.11	(−0.32, 0.08)	0.241
Serum aspartate aminotransferase (AST)	0.06	(−0.07, 0.19)	0.379	0.09	(−0.08, 0.23)	0.325	−0.02	(−0.25, 0.21)	0.846
Serum γ-glutamyltranspeptidase (γ-GTP)	0.19	(0.06, 0.33)	0.006	0.28	(0.09, 0.47)	0.005	0.12	(−0.09, 0.31)	0.268
Serum uric acid (UA)	0.05	(−0.07, 0.18)	0.393	−0.04	(−0.20, 0.13)	0.674	0.08	(−0.11, 0.26)	0.432
Serum creatinine	0.002	(−0.12, 0.13)	0.979	0.04	(−0.13, 0.21)	0.627	0.02	(−0.17, 0.20)	0.849
White blood cells	0.31	(0.19, 0.43)	<0.001	0.39	(0.22, 0.55)	<0.001	0.17	(−0.02, 0.35)	0.081
Height	0.18	(0.06, 0.29)	0.003	0.28	(0.11, 0.39)	0.001	0.03	(−0.16, 0.23)	0.717

β: Standardized parameter estimate calculated by multiple linear regression analysis. CI: Confidence interval. TG, γ-GTP and serum creatinine are calculated in logarithm values.

We also evaluated the correlation between height and reticulocytes limited to elderly subjects (Age≥67) (n=140), and found that the significant correlation became slightly stronger: β=0.26 (0.11, 0.40), p=0.001 for total subjects, β=0.31 (0.08, 0.47), p=0.006 for subjects with a high hemoglobin concentration (n=72), and β=0.21 (−0.03, 0.48), p=0.084 for subjects with a low hemoglobin concentration (n=68).

## DISCUSSION

The main finding of the present study was a significant positive correlation between height and reticulocyte in elderly Japanese men, especially in subjects with a high hemoglobin concentration, independent of known cardiovascular risk factors and other hematological parameters (WBC).

In a previous study, we reported an inverse association between height and normocytic normochromic anemia in Japanese men [10]. Since normocytic normochromic anemia might be caused by reduced productivity of hemoglobin in the bone marrow, we surmised that height might indicate hematopoiesis. However, a wide range of age groups (40-89 years) were employed in that study, which might have acted as a strong confounding factor with for the correlation between height and anemia and obscured the presence of any direct hematopoiesis. The present study employed subjects in a narrow age range (65-69 years) that showed no significant correlation with height (r=-0.11, p=0.088), and used reticulocyte, which directly indicates hemoglobin productivity.

However, the mechanisms underlying the positive correlation between reticulocyte and height among elderly men are not yet clear. The side population of hematopoietic stem cells in the bone marrow decreases as individuals age [18,19], which may be associated with increased frequency of anemia seen in the elderly [20]. Neumann reported a decline in active hematopoietic (red) bone marrow with age and transformation into fatty (yellow) marrow from the periphery towards the axial skeleton [13]. Since the volume of bone marrow is smaller in subjects with a shorter stature compared to those with a taller stature, any reduction in the capacity of the hematopoietic bone marrow could be of crucial importance to individuals in the former group. In our additional analysis limited to elderly subjects (age≥67), the slightly stronger correlation between height and reticulocytes among total subjects and subjects with a high hemoglobin concentration might support the above-mentioned mechanism.

We also found that the significant positive correlation between height and reticulocytes is limited to subjects with a high hemoglobin concentration. Since hemo-globin is independently positively associated with hypertension [15], hypertension-induced vascular damage [16], and atherosclerosis [17], subjects with a high hemoglobin concentration should have higher hematogenesis activity and vascular repair than subjects with low a hemoglobin concentration. Therefore, the analysis limited to subjects with a high hemoglobin concentration should emphasize hematopoietic capacity since the productivity of reticulocytes could increase by a limited level in elderly subjects.

Possible limitations of this study warrant consideration. Because creatinine clearance data were not available and estimated glomerular filtration rate (GFR) is not an effective tool for evaluating kidney function for a comparison of associations with various body heights [10,21,22], we could not perform an analysis adjusted for exact renal function. However, our study showed that the correlation between height and reticulocyte remained significant even after adjustment for serum creatinine. Next, because data for serum iron and vitamin concentrations were not available as well, we were not able to evaluate the influence of these factors. Although height is significantly positively correlated with reticulocytes in elderly Japanese men with a normal BMI, we were not able to conduct meaningful statistical analyses for subjects with an abnormal BMI status (e.g., low (<18.5kg/m2) or high (≥25.0kg/m2)) due to the limited number of participants. Since 25.6% subjects had a high BMI and 12.2% subjects had a low BMI in our present study, the influence of these subjects on the general population is tangible. However, abnormal BMI status might act as a strong confounding factor on the correlation between height and reticulocytes. Therefore, to evaluate the correlation between height and reticulocytes in these subjects, analyses with a larger population and stratification by BMI status should be conducted. Finally, because this was a cross-sectional study, causal relationships were not able to be established.

In conclusion, a significant positive correlation between height and reticulocyte was seen in elderly Japanese men. Height could act as a surrogate marker of hemato-poiesis in the elderly. Since short stature is reported to be correlated with a high mortality and/or incidence of cardiovascular disease [1–10], and anemia reflects poor health and increased vulnerability to poor outcomes in older persons [11,12], these results might represent an efficient tool to clarify the underlying mechanism of risk for short stature.

## MATERIALS AND METHODS

### Study population

To avoid the influence of age on height, this study was comprised of subjects in a narrow age range. The original population included 409 men 65 to 69 years old residing in rural communities in Nagasaki Prefecture in western Japan. Participants were recruited in 2013-2015. To avoid the influence of inflammatory and hematological disease, subjects with high and low white blood cell count (≥10,000 cells/μL (n=2) and 1,000 cells/μL< (n=1), respectively) were excluded. Also, to avoid the influence of medication activating the bone marrow, subjects taking medication for anemia (n=3) were excluded.

Since hemoglobin value shows a strong positive correlation with body mass index [15,17], and another study reported a J- or U-shaped correlation between BMI and mortality [23], abnormal BMI status might act as a strong confounding factor for the correlation between height and reticulocyte. Therefore, to avoid the influence of undernutrition and hypernutrition, subjects with a BMI<18.5kg/m^2^ (n=50) and BMI≥25kg/m^2^ (n=103), respectively, were excluded. Subjects with no evaluable laboratory data (n=1) were also excluded, leaving a total of 249 subjects participating in the study. Written consent forms were available in Japanese to ensure comprehensive understanding of the study objectives, and informed consent was signed by the participants. This study was approved by the Ethics Committee for Human Use of Nagasaki University (project registration number 0501120073).

### Data collection and laboratory measurements

Trained interviewers obtained information on medical history. Body weight and height of patients wearing light clothing were measured using an automatic body composition analyzer (BF-220; Tanita, Tokyo, Japan), and body mass index (BMI; kg/m^2^) was calculated.

Fasting blood samples were collected in an EDTA-2K tube and a siliconized tube. Samples from the EDTA-2K tube were used to measure white blood cell count (WBC) and reticulocyte using the flow cytometry method at SRL, Inc. (Tokyo, Japan). Serum triglyceride (TG), serum high density lipoprotein (HDL) cholesterol, serum aspartate aminotransferase (AST), serum γ-glutamyltranspeptidase (γ-GTP), hemoglobin A1c (HbA_1C_), serum uric acid, and serum creatinine were measured using standard laboratory procedures at SRL, Inc. (Tokyo, Japan).

### Statistical analysis

Characteristics of the study population stratified by hemoglobin levels concentration were expressed as mean ±standard deviation. Simple and partial correlation analysis adjusted for known cardiovascular risk factors and WBC were performed to evaluate reticulocytes and other existing parameters. We also performed simple and multiple linear regression analysis to evaluate the same. Since intercorrelation with systolic blood pressure was r= 0.73 (P<0.001), diastolic blood pressure was not analyzed as a confounding factor. Because TG, γ-GTP, and serum creatinine had a skewed distribution, logarithmic transformation was performed for the simple and partial correlation analysis, and linear regression analysis. All statistical analyses were performed with the SAS system for Windows (version 9.4; SAS Inc., Cary, NC). Probability values of less than 0.05 were considered to be statistically significant.
